# Tenofovir disoproxil fumarate induces peripheral neuropathy and alters inflammation and mitochondrial biogenesis in the brains of mice

**DOI:** 10.1038/s41598-019-53466-x

**Published:** 2019-11-20

**Authors:** Jerel Adam Fields, Mary K. Swinton, Aliyah Carson, Benchawanna Soontornniyomkij, Charmaine Lindsay, May Madi Han, Katie Frizzi, Shrey Sambhwani, Anne Murphy, Cristian L. Achim, Ronald J. Ellis, Nigel A. Calcutt

**Affiliations:** 1 Department of Psychiatry, University of California San Diego, La Jolla, CA USA; 2 Department of Neurosciences, University of California San Diego, La Jolla, CA USA; 3 Department of Pathology, University of California San Diego, La Jolla, CA USA; 4 Department of Pharmacology, University of California San Diego, La Jolla, CA USA

**Keywords:** Cellular neuroscience, Molecular neuroscience

## Abstract

Mounting evidence suggests that antiretroviral therapy (ART) drugs may contribute to the prevalence of HIV-associated neurological dysfunction. The HIV envelope glycoprotein (gp120) is neurotoxic and has been linked to alterations in mitochondrial function and increased inflammatory gene expression, which are common neuropathological findings in HIV+ cases on ART with neurological disorders. Tenofovir disproxil fumarate (TDF) has been shown to affect neurogenesis in brains of mice and mitochondria in neurons. In this study, we hypothesized that TDF contributes to neurotoxicity by modulating mitochondrial biogenesis and inflammatory pathways. TDF administered to wild-type (wt) and GFAP-gp120 transgenic (tg) mice caused peripheral neuropathy, as indicated by nerve conduction slowing and thermal hyperalgesia. Conversely TDF protected gp120-tg mice from cognitive dysfunction. In the brains of wt and gp120-tg mice, TDF decreased expression of mitochondrial transcription factor A (TFAM). However, double immunolabelling revealed that TFAM was reduced in neurons and increased in astroglia in the hippocampi of TDF-treated wt and gp120-tg mice. TDF also increased expression of GFAP and decreased expression of IBA1 in the wt and gp120-tg mice. TDF increased tumor necrosis factor (TNF) α in wt mice. However, TDF reduced interleukin (IL) 1β and TNFα mRNA in gp120-tg mouse brains. Primary human astroglia were exposed to increasing doses of TDF for 24 hours and then analyzed for mitochondrial alterations and inflammatory gene expression. In astroglia, TDF caused a dose-dependent increase in oxygen consumption rate, extracellular acidification rate and spare respiratory capacity, changes consistent with increased metabolism. TDF also reduced IL-1β-mediated increases in IL-1β and TNFα mRNA. These data demonstrate that TDF causes peripheral neuropathy in mice and alterations in inflammatory signaling and mitochondrial activity in the brain.

## Introduction

Combination antiretroviral therapy (ART) has extended the life expectancy of people with HIV (PWH)^1–3^. However, comorbidities such as peripheral neuropathy and cognitive dysfunction persist in people in whom ART has suppressed viral loads^1,4–6^. As the population of PWH ages and people of older age are more often newly infected compared to early on in the epidemic^7^, there is a need to better understand how ART drugs and low-level viral replication or protein production may contribute to comorbidities in PWH.

Despite the success of ART, neurological disorders of the peripheral nervous system (PNS) and central nervous system (CNS) affect approximately 20–50% of PWH^1,8,9^. In the pre-ART era HIV associated neurocognitive disorders (HAND) affected as high as 50% of PWH^10–12^. However, the severity of HAND has been reduced with only 2–3% of PWH being afflicted with HIV-associated dementia^10^. The majority of HAND cases are diagnosed with asymptomatic neurocognitive impairment (ANI) or minor neurocognitive disorder (MND)^10^. The high prevalence of the more subtle categories of HAND is at first glance confounding since PWH on ART often have low to undetectable viral loads. Studies have shown that low-level HIV protein expression may persist in PWH on ART^13,14^, but mounting evidence suggest that ART drugs may also underlie some of the neurological dysfunction^15–20^.

Nucleoside reverse transcriptase inhibitors (NRTI) have been linked to mitochondrial dysfunction since soon after their implementation^21,22^. AZT and d-drugs were associated with mitochondrial pathology in peripheral neurons^21,22^. Mitochondrial DNA (mtDNA), a key component of mitochondrial biogenesis and gene expression, may be damaged by ART drugs^21,23^. Initial studies determined that NRTI drugs altered the function of human mtDNA polymerase γ and damaged mtDNA^21,24^. Reductions in metabolites such as acylcarnitine in the brain suggested that mitochondrial dysfunction may persist in people on ART^25,26^. Newer ART regimens do not show the same level of toxicity as older drugs. However, symptoms of neuronal dysfunction persist in patients starting on newer drugs. The NRTI, tenofovir disoproxil fumarate (TDF), a highly prevalent component of modern ART regimens, has been to shown to affect mitochondria in cultured neurons^20^. *In vivo* mouse studies have shown that TDF can affect neurogenesis in the brain, which could be involved in the persistence of HAND^16^. TDF is part of a combination of ART drugs taken as preventative prophylaxis by people at high risk for contracting HIV. It is important to understand if TDF is toxic to mitochondria in the CNS *in vivo*.

Mitochondrial biogenesis is increasingly seen as a therapeutic target in both peripheral neuropathy and CNS neurodegenerative diseases^27^. To maintain healthy mitochondria, under a tightly regulated process, cells generate new mitochondria, while degrading damaged mitochondria (mitophagy)^28^. Mitochondrial biogenesis requires activation of transcription factors peroxisome proliferator-activated receptor α coactivator 1-α (PGC-1α) and mitochondrial transcription factor A, (TFAM) when energy (adenosine triphosphate [ATP]) is low. Alterations in levels of PGC-1α and TFAM are implicated in several neurodegenerative diseases including Alzheimer’s Disease (AD), Huntington’s disease (HD), and Parkinson’s disease (PD)^29–32^. HIV infection^33^, ART^34^, and AD are also associated with reduced levels mtDNA in the brain^35^. Remarkably, enhancing mitochondrial biogenesis^32,36^ is neuroprotective in rodent models of AD, HD, and PD. We recently reported that overall levels of PGC-1α and TFAM in the frontal cortex of HAND brains are reduced, particularly in neurons^37^. Interestingly, astroglia have increased levels of TFAM in these same brains. Increased astroglial TFAM may be due to activation by inflammatory cytokines, though the precise mechanism of this shift in HAND brains is not well understood^37^.

Although viral replication is low in PWH on ART, there is some evidence that HIV protein production persists in both the periphery and in the CNS^13,14^. Astrogliosis and microgliosis remain a hallmark of HIV infection in the ART era^38,39^. Increased levels of inflammatory gene expression are still observed in postmortem brain specimens from people that were on ART^40^. Though there are no perfect models to study HIV infection and much less HIV infection during the ART era, the GFAP-gp120 transgenic (gp120-tg) mouse model reproduces much of the neuropathology observed in postmortem brains from HAND cases^41,42^. The gp120 tg model shows increased astrogliosis and microgliosis and reduced integrity of neuronal processes and synapses, mirroring reports in HAND brains from the ART era^17,41,43^. More detailed neuropathological hallmarks of HAND have also been observed in the gp120-tg mouse model including alterations in autophagy, and mitochondrial dynamics and morphology^41,42^. The gp120 tg mouse model may provide a solid platform to investigate the combined effects of some HIV protein expression in the context of ART exposure.

In the present study, wild-type (wt) and gp120 tg mice were utilized to understand the effects of TDF and gp120 on PNS function and on mitochondrial biogenesis transcription factors PGC-1α and TFAM and mitochondrial fission protein, dynamin-like protein (DNM1L). We also performed neuropathological analyses to understand how alterations in mitochondrial biogenesis are associated with neurodegeneration, gliosis and inflammation. Finally, we corroborated our findings using *in vitro* models for human astroglia and microglia. The data herein provide new details on how TDF alters mitochondrial biogenesis and inflammatory signaling in *in vivo* and *in vitro* settings.

## Methods

### Mouse studies

For these studies, an animal model of HIV-protein mediated neurotoxicity, tg mice expressing high levels of gp120 under the control of the glial fibrillary acidic protein (GFAP) promoter, were used as previously described^41,44^. These mice develop neurodegeneration accompanied by astrogliosis, microgliosis, and memory deficits in the water maze test^44^. They have also been reported to develop indices of peripheral neuropathy after 12–15 months of age, onset of which can be accelerated by treatment with the reverse transcriptase inhibitor didanosine^45^. Wt and gp120-tg mice were aged 7–8 months at the start of the study. TDF was purchased from MedChemExpress LLC and dissolved in sterile H_2_O at 6 mg/ml. Mice were treated with vehicle or TDF (50 mg/kg) daily by oral gavage for 4 weeks. Mice underwent assays for sensorimotor coordination, learning and memory (repeated accelerating rotarod test^46,47^), paw thermal sensation and large fiber motor nerve conduction velocity (MNCV), exactly as described in detail elsewhere^48^ with assays performed both before and after the treatment regimen. After physiological and behavioral testing was complete, mice were euthanized and brains were excised 3 days after last administration of TDF, sagittally sectioned and the left hemibrain was snap frozen for biochemical and RNA analyses and the right hemibrain was immersion immersion fixed in 4% paraformaldehyde for one week.

### Rotarod

Each mouse was placed on a rotating rod (1.25 inch diameter: Rotarod, Stoelting) facing the experimenter. Rotation was activated, and the rate of rotation increased gradually from the starting speed of 4 rotations per minute (RPM) to a maximum of 40 RPM within 120 seconds. The time at which the mouse fell from the rod was recorded by a sensor.

### Motor nerve conduction velocity

To evaluate the function of large myelinated motor nerve fibers, motor nerve conduction velocity (MNCV) was measured as described in detail elsewhere^48^. Briefly, mice anesthetized with 4% isoflurane in oxygen and four platinum-tipped sub-dermal needle electrodes (Grass Technologies) connected to a PowerLab stimulator (AD Instruments). The grounding electrode was inserted into skin at the back of the neck. One of the recording electrodes was inserted into the interosseous muscle between the second and third toes, and the other into the muscle between the third and fourth toes. The PowerLab stimulator was set to deliver a 200-mV, 50-μsec-duration square wave stimulus every 2 seconds. The stimulating electrode was first inserted into the ankle at the Achilles tendon and the resulting electromyogram (EMG) containing the M wave (M Achilles wave) recorded. The stimulating electrode was moved to the sciatic notch and the EMG M wave at the notch (M notch wave) recorded. This process was repeated three times while alternating the stimulating electrode between the Achilles tendon and the sciatic notch. The leg was then stretched and the superficial distance between the Achilles tendon and the sciatic notch measured with a caliper. To calculate MNCV in m/s, the difference between M Achilles and M notch was calculated for all three repeats and the median value divided by the distance between the Achilles tendon and the sciatic notch.

### Heat sensitivity testing

The function of small sensory fibers in paw skin was measured as described in detail elsewhere^48^ using a Hargearves test device (UARD) to record time to paw withdrawal from a heat stimulus that increased at a rate of 1 °C/sec from a starting surface temperature of 30 °C. Mice were placed on the glass surface of the device inside restraint chambers and allowed an acclimation period of 15 minutes. After acclimation, the heat source was moved directly under the plantar surface of the left hind paw and activated. A movement sensor detected paw withdrawal from the glass surface, stopped the heating and recorded the time between onset of heating and paw withdrawal. Heating automatically stopped after 20 seconds (50 °C) to prevent paw damage. Measurements were not made when mice were grooming, urinating or rearing, only when the paws remained flat on the glass surface. Measurements were made on both hind paws and repeated four times. The median of the four measurements for each hind paw was calculated and thermal response latency of the mouse calculated by averaging the median measurements of left and right hind paws. The thermal response latency (seconds) was converted to response temperature using a calibration curve that was constructed from multiple daily measurements of the heating rate of the glass surface.

### Immunoblot

Mouse brains were homogenized and fractionated using a buffer that facilitates separation of the membrane and cytosolic fractions (1.0 mmol/L HEPES (Gibco, cat. no. 15630–080), 5.0 mmol/L benzamidine, 2.0 mmol/L 2-mercaptoethanol (Gibco, cat. no. 21985), 3.0 mmol/L EDTA (Omni pur, cat. no. 4005), 0.5 mmol/L magnesium sulfate, 0.05% sodium azide; final pH 8.8) as described in a previous publication^37^. In brief, as previously described^42^, tissues from brain samples (0.1 g) were homogenized in 0.7 ml of fractionation buffer containing phosphatase and protease inhibitor cocktails (Calbiochem, cat. no. 524624 and 539131). Samples were precleared by centrifugation at 5000 × g for 5 min at room temperature. Supernatants were retained as the whole lysate and stored at −80 until use.

As previously described^37^, after determination of the protein content of all samples by bicinchoninic acid assay (Thermo Fisher Scientific, cat. no. 23225) and denaturing in lamellae sample buffer (Bio Rad, cat. no. 1610747), whole lysates were loaded (10 μg total protein/lane) on 4–15% Criterion TGX stain free gels (Bio Rad, cat. no. 5678085) and electrophoresed in Tris/Glycine/SDS running buffer (Bio Rad, cat. no. 161–0772) and transferred onto LF PVDF membrane with Bio Rad transfer stacks and transfer buffer (Bio Rad, cat. no 1704275) using Bio Rad Trans Blot Turbo transfer system. After the transfer, total protein was imaged using Bio Rad ChemiDoc imager under the stain free blot setting for normalization purposes. The membranes were then blocked in 1% casein in tris-buffered saline (TBS) (Bio Rad, cat. no. 1610782) for 1 h. Membranes were incubated overnight at 4 °C with primary antibodies diluted in blocking buffer. All blots were then washed in PBST, and then incubated with species-specific IgG conjugated to HRP (American Qualex, cat. no. A102P5) diluted 1:5000 in PBST and visualized with SuperSignal West Femto Maximum Sensitivity Substrate (ThermoFisher Scientific, cat. no. 34096). Images were obtained, and semi-quantitative analysis was performed with the ChemiDoc gel imaging system and Quantity One software (Bio-Rad).

### Immunohistochemistry and double immunolabeling of brain sections

As previously described^37^, free-floating 40 μm thick vibratome sections of mouse brains were washed with phosphate buffered saline with tween 20 (PBST) 3 times, pre-treated for 20 minutes in PBS 3% H_2_O_2_/1%TritonX, and blocked with 2.5% horse serum (Vector Laboratories) for 1 hour at room temperature. Sections were incubated at 4 C overnight with the primary antibodies, TFAM (Invitrogen, cat. no. PA5-23776), GFAP (Sigma, cat. No. G3893), and IBA1 (Wako, cat. no. 019–19741). Sections were then incubated in secondary antibody, Immpress HRP Anti-rabbit IgG (Vector, cat. no. MP-7401) or Impress HRP Anti-mouse IgG (Vector, cat. No. MP-7402) for 30 minutes, followed by NovaRED Peroxidase (HRP) Substrate made with NovaRED Peroxidase (HRP) Substrate Kit as per manufacturer’s instructions (Vector, cat. no. SK-4800). Control experiments consisted of incubation with secondary antibody only. Tissues were mounted on Superfrost plus slides and coverslipped with cytoseal. Immunostained sections were imaged with a digital Olympus microscope and assessment of levels of TFAM, GFAP, and IBA1 immunoreactivity was performed utilizing the Image- Pro Plus program (Media Cybernetics, Silver Spring, MD). For each case a total of three sections (10 images per section) were analyzed in order to estimate the average number of immunolabelled cells per unit area (mm^2^) and the average intensity of the immunostaining (corrected optical density). Background levels were obtained in tissue sections immunostained in the absence of primary antibody. Therefore: corrected optical density = optical density − background. GFAP and IBA1 positive cells were quantified in the hippocampi using Image J analysis software.

Double immune-labeling was performed in the same manner as immunohistochemistry staining up until secondary antibody incubation. Sections were stained with primary antibodies, TFAM, GFAP, MAP2 and IBA1 overnight at 4 degrees C, after which they were washed three times with PBS and incubated in secondary fluorescent antibodies for 30 min. After incubating in secondary antibodies, tissues were washed with PBST three times and then incubated in DAPI diluted 1:10,000 for 15 min. After 15 min, DAPI was removed and tissues were washed three times with PBST before mounting on coverslips and cover slipping with vectashield.

### RNA isolation and real-time reverse transcription polymerase chain reaction (RT^2^PCR)

RNA was isolated from the frontal cortex of mouse brains using the RNeasy Lipid Tissue Kit (Qiagen, cat. No. 74804). Brain tissue was homogenized in Qiazol followed by the addition of chloroform. Homogenates were centrifuged at 12,000 g for 15 min. The aqueous layer was removed, and RNA was isolated from this layer according to manufacturers’ instructions.

As previously described^37^, RNA was reverse transcribed into cDNA with a High capacity cDNA Reverse Transcription Kit (Life technologies, cat. no. 4358813) as per manufacturer’s instructions. Gene expression was determined using rt^2^PCR TaqMan gene expression assays performed using the StepOnePlus sequence-detection system (Life Technologies), using primers specific to IL-1β and TNFα. ACTB was used as a normalization control. A master mix was made using 2x Fast advanced master mix (Thermofisher, cat. no. 4444557), 20x primers, and water. 8ul of master mix and 2ul of cDNA was added to each reaction well (The reactions were carried out at 48 °C for 30 min and 95 °C for 10 min, followed by 40 cycles of 95 °C for 15 s and 60 °C for 1 min). Samples were analyzed in duplicate. Fold changes were calculated using the comparative C_T_ method.

### Cell culture

This study was approved by the University of California San Diego Human Research Protections Program. All research was performed in adherence to the relevant guidelines and regulations. As previously described^37^, astroglia were isolated from fetal human brain tissue from elective terminated pregnancy between 12 and 16 weeks of gestation, acquired from Advanced Bioscience Resources. Donors gave written informed consent for research-use of the cells and tissue. Tissue was fragmented and mechanically dissociated using a scalpel and washed 3 times with HBSS holding media (Gibco, cat. no.14175–095) with 1 mM Glutamax (Gibco, cat. no. 35050–061), 20ug/mL Gentamicin (Gibco, cat. no. 15710–064) and 5 mM HEPES (Gibco, cat. no. 15630–080). The tissue was homogenized with the addition of 15 mL of 0.25% trypsin EDTA (Gibco, cat. no. 25200–056) for 5 min in a 37 °C incubator. After 5 min, 1 mL of a trypsin inhibitor (Roche, cat. no. 10109) and 24 mL of DMEM media (Gibco, cat. no. 11960–044) with human serum (Corning, cat. no. 35–060-cl) was added. The mixture was then centrifuged for 5 min at 4 °C to pellet the cells. Supernatant was removed and discarded, and the cells were resuspended in 5 ml of DMEM media and strained with a 70 μM strainer (Falcon, cat. no. 352350). The cell suspension was underlaid with 7 ml of a solution of filtered 8% BSA in PBS and cells were centrifuged at 1 × 10^4^ rpm at 4 °C for 10 min. The supernatant was removed, and the cells were resuspended in DMEM media with human serum. Astroglia were plated at a density of 1 × 10^7^/T75 flask and cultured as adherent monolayers. After 1 week, the astroglia DMEM media with human serum was replaced with DMEM media with 10% fetal bovine serum (FBS) (Gibco, cat. no. 16000044) and 1% penicillin/ streptomycin (P/S) (Corning, cat. no. 30–001-CI-1). Every 3 days, a half media exchange was performed on each cell type.

A vial of human microglial cell 3 (HMC3) was purchased from (CRL-3304). HMC3 and cultured in EMEM with 10% FBS and penicillin/streptomycin as per ATCC instructions. Cells were split every 5 days using trypsin-EDTA.

### Antibodies

GFAP (Sigma Aldrich, cat# G3893), TFAM (Invitrogen, cat# PA5-23776) and MAP2 (Santa Cruz Biotechnologies, cat# sc-32791), PGC-1α (Abcam, cat#. ab54481), IBA1 (Wako, cat# 019-19741).

### Seahorse assay

As previously described^37^, astroglia were split into a seahorse plate at 3 × 10^4^ cells/well and treated with Vehicle or TDF for 24 h. On the following day, cultures were incubated in a non-CO_2_ incubator at 37 °C to equilibrate for approximately 30 minutes prior to assay. Baseline measurements of ECAR were taken prior to addition of oligomycin (2 μM), followed by a titrated concentration of FCCP, and then rotenone (500 nM) together with antimycin (1 μM) (Sigma-Aldrich, cat no. A8674). After each addition of mitochondrial inhibitor, three readings were taken before injection of the subsequent inhibitor. ECAR was automatically calculated and recorded by the Seahorse XFe96 software. Rates were calculated by the Seahorse analyzer, reported as log of H^+^ production rate and then normalized to control for presentation. Samples were run in biological replicates of five in two independent experiments.

### Ethical approval for studies

This study was approved by the University of California San Diego Human Research Protections Program. All experiments described were approved by the animal subjects committee at the University of California, San Diego (UCSD), and were performed according to NIH recommendations for animal use.

## Results

### Indices of peripheral neuropathy are induced by Tenofovir disproxil fumarate but cognitive performance is protected

WT and gp120-tg mice were indistinguishable at onset of the study, as indices of peripheral neuropathy do not develop in this strain until around 1 year of age^44^. TDF induced significant MNCV slowing and paw thermal hyperalgesia in both wt and gp120-tg mice compared to pre-treatment values (Table 1). We also measured rotarod performance, both as an indicator of sensorimotor function when measured in isolation and also as an index of learning and memory when measured iteratively^46,47^. All groups showed similar performance at baseline whereas at study end, WT mice treated with vehicle or TDF spent significantly more time on the rotarod, indicative of learning and memory (Table 1). Vehicle-treated gp120-tg mice did not improve performance over time, whereas significant improvement was restored in gp120-tg mice treated with TDF.

**Table 1:** Peripheral neuropathy caused by tenofovir (T).

Group	N	M:F	Body Weight (g)		Rotarod Latency (sec)		MNCV (m/sec)		Heat Response Threshold (°C)	
			Onset	Final	Onset	Final	Onset	Final	Onset	Final
WT + V	8	1:7	28.5 ± 1.9	27.8 ± 2.0	11.1 ± 1.9	18.9 ± 3.5^a^	43.2 ± 1.4	41.1 ± 2.9	38.8 ± 0.6	38.0 ± 0.7
WT + T	5	2:3	31.4 ± 3.7	30.8 ± 4.2	8.1 ± 0.7	17.6 ± 3.4^a^	44.1 ± 0.7	28.8 ± 5.2^a^	39.8 ± 1.0	36.0 ± 0.5^a^
gp120-tg + V	9	6:3	31.4 ± 2.5	30.2 ± 2.5	10.4 ± 1.1	11.4 ± 3.4	42.1 ± 1.8	38.9 ± 3.7	40.9 ± 1.0	39.8 ± 1.3
gp120-tg + T	12	8:4	29.6 ± 1.9	28.7 ± 2.1	9.7 ± 1.1	18.2 ± 1.8^c^	43.7 ± 2.0	32.7 ± 3.2^c^	40.2 ± 0.6	37.7 ± 0.7^b^

Mice were 7–8 months old at the start of the study and tenofovir was given daily (50 mg/kg po) for 4 weeks. Data are group mean ± SEM. ^a,b,c^p < 0.05, p < 0.01 and p < 0.001 vs group onset values by paired t test.

### PGC-1α, TFAM and DNM1L protein levels are altered in brain lysates from wt and gp120-tg mice administered Tenofovir disproxil fumarate

**Figure 1:**
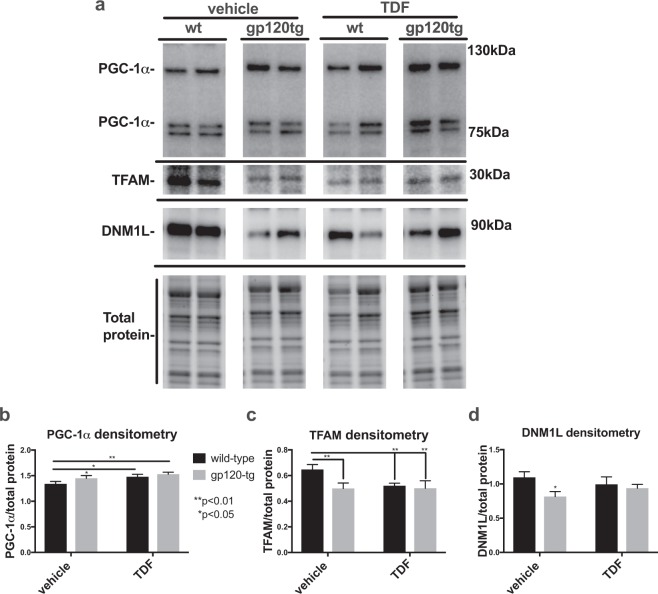
PGC-1α protein levels are increased and TFAM and DNM1L protein levels are decreased in brain lysates from wt and gp120-tg mice administered Tenofovir disproxil fumarate. (**a**) Immunoblot for PGC-1α, TFAM, and DNM1L using whole lysates from hippocampal region. (**b—d**) Densitometry for quantification of PGC-1α, and TFAM, and DNM1L normalized to total protein levels. Significance (p < 0.05) was determined by two-way ANOVA (wt, n = 8; wt + TDF, n = 5; gp120-tg, n = 7; gp120-tg, n = 7).

To determine the relative abundance of key transcription factors involved in mitochondrial biogenesis in mouse brains, whole lysates were assayed for PGC-1α, TFAM, and DNM1L levels by immunoblot. The band corresponding to PGC-1α appeared as a single band in the expected 100 kDa range, with the most intense and large band appearing in the gp120-tg mice treated with TDF (Fig. 1a). The raw immunoblot images are included (Supplementary Fig. 1). The signal for TFAM appeared at the 30 kDa mark in a similar pattern, with the strongest band for TFAM detected in the wt mice (Fig. 1a). The band for DNM1L appeared at 90 kDa with the strongest band in the wt mice, weakest in the gp120tg mice (Fig. 1a). Densitometry analyses were performed on each band by normalizing to the total protein transferred to the PVDF membrane for that lane. The densitometry for PGC-1α was significantly (**p < 0.01) increased in TDF treated, gp120-tg and in gp120-tg + TDF compared to wt (Fig. 1b). Densitometry analyses showed the signal for TFAM was significantly (**p < 0.01) reduced in TDF treated, gp120-tg and in gp120-tg + TDF compared to wt (Fig. 1c). Densitometry analyses for DNM1L bands confirmed a significant reduction in gp120-tg mice compared to wt (Fig. 1d). However, DNM1L in gp120-tg mice treated with TDF was not significantly different than wt or wt + TDF (Fig. 1d).

### TFAM and MAP2 signals are reduced in hippocampi from wt and gp120-tg mice administered Tenofovir disproxil fumarate

**Figure 2:**
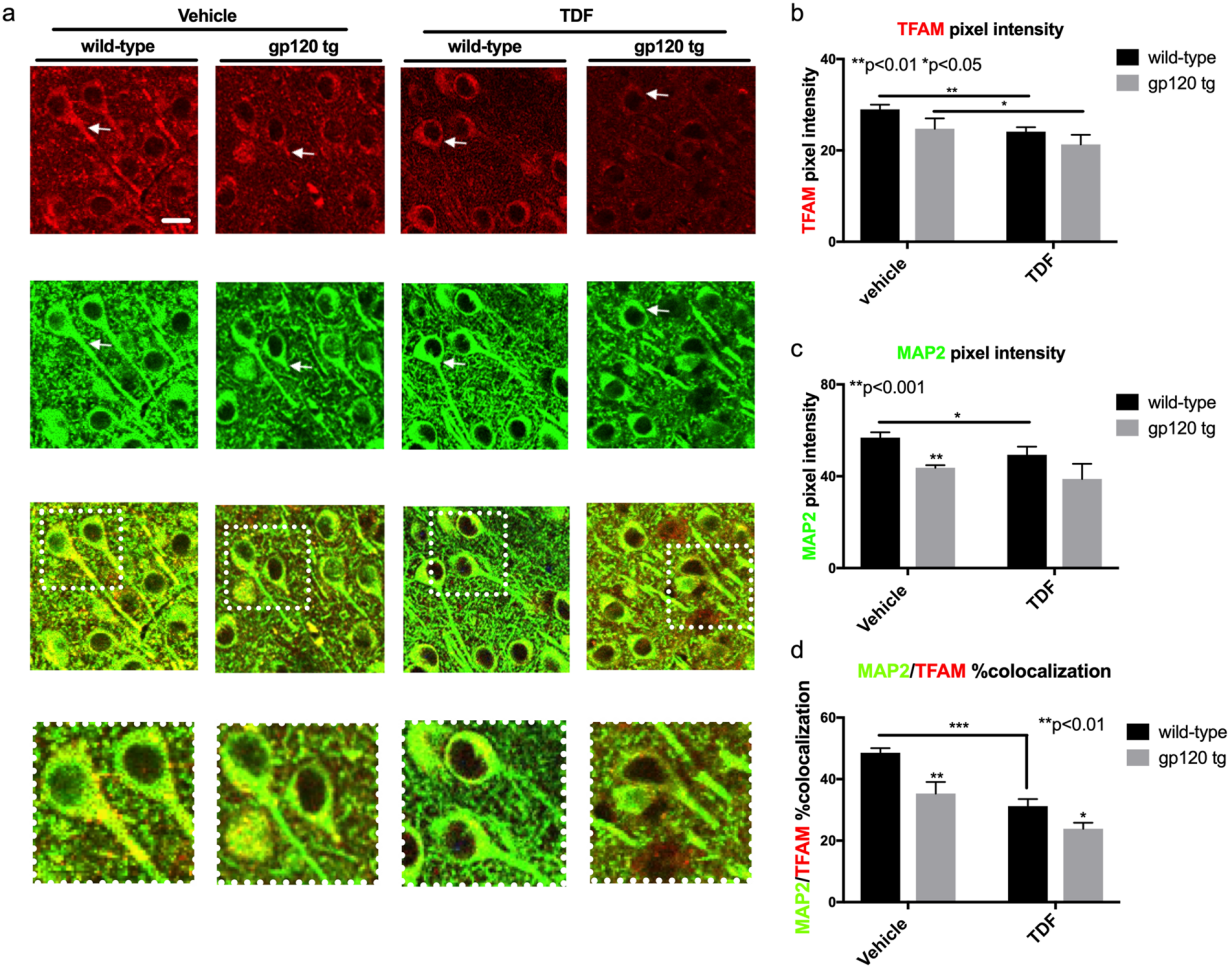
TFAM and MAP2 signals are reduced in hippocampi from wt and gp120-tg mice administered Tenofovir disproxil fumarate. (**a**) Vibratome sections of mouse brains were double immunolabelled with antibodies for TFAM and MAP2. (**b**) MAP2 pixel intensity was quantified using Image J software. (**d**) The percent of the TFAM signal (red) colocalizing with the MAP2 signal (green) was assessed using Image J and the SQUASSH plug-in (white arrows indicate points of colocalization). Significance (p < 0.05) was determined by two-way ANOVA (wt, n = 3; wt + TDF, n = 4; gp120-tg, n = 4; gp120-tg, n = 4).

In order to determine the neuronal integrity in the brains of wt and gp120-tg mice treated with TDF, we double immunolabeled vibratome sections with TFAM (red) and MAP2 (green). In brains from vehicle treated wt mice the signal for TFAM was present throughout the soma and processes of hippocampal neurons (CA1 region) (Fig. 2a). However, in vehicle-treated gp120-tg or wt and gp120-tg mice treated with TDF, the TFAM signal was more punctate throughout the soma and processes of neurons (Fig. 2a). MAP2 signal was as expected in wt mice, being noted throughout neuronal soma and processes, but in vehicle-treated gp120-tg and gp120-tg mice treated with TDF the MAP2 signal was greatly diminished suggesting loss of neuronal integrity (Fig. 2a). Yellow signal, indicating colocalization of MAP2 with TFAM, was noticeably reduced in all three groups, in vehicle-treated gp120-tg or wt and gp120-tg mice treated with TDF, compared to the brains of vehicle-treated wt mice (Fig. 2a). These observations were supported by quantification of pixel intensity in the confocal images. In mouse brains from vehicle-treated gp120-tg or wt and gp120-tg mice treated with TDF, the signal intensity for TFAM was significantly decreased compared to wt mouse brains (Fig. 2b). In mouse brains from vehicle-treated gp120-tg or wt and gp120-tg mice treated with TDF, the MAP2 pixel intensity was significantly decreased (Fig. 2c). In mouse brains from vehicle treated in vehicle-treated gp120-tg or wt and gp120-tg mice treated with TDF, the percent of TFAM signal colocalizing with MAP2 signal was significantly decreased (Fig. 2d).

### GFAP and GFAP/TFAM colocalization is increased in GP120 mouse hippocampi

**Figure 3:**
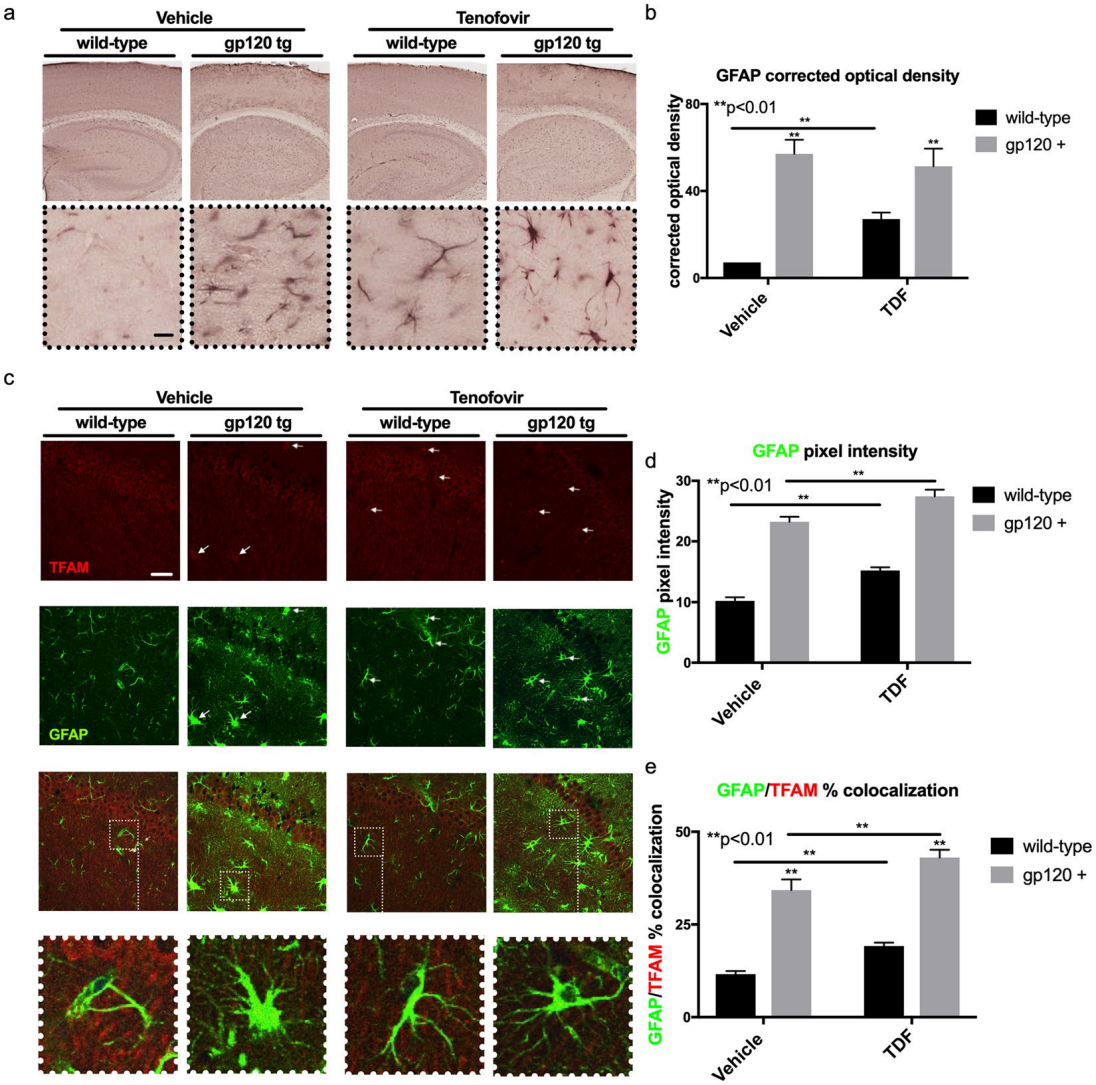
Tenofovir disproxil fumarate increases GFAP expression, reduces TFAM expression, and increases TFAM colocalization with GFAP in hippocampi of wt and gp120tg mice. Vibratome sections were immunolabelled with antibody against GFAP and then double immunolabelled for TFAM and GFAP. (**a**) GFAP-DAB immunostaining in hippocampi. (**b**) Quantification of GFAP corrected optical density. (**c**) TFAM (red) and GFAP (green) signal in hippocampi. (**d–e**) Quantified colocalization of TFAM and GFAP and colocalization of TFAM with GFAP (white arrows indicate points of colocalization). Analyzed with two-way ANOVA test (wt, n = 3; wt + TDF, n = 4; gp120-tg, n = 4; gp120-tg, n = 4).

Astrogliosis can be identified by immunostaining astroglia for glial fibrillary acidic protein (GFAP). Due to the elongation of astroglia processes, astrogliosis will present with an increase in staining for GFAP compared to the inactivated state. To determine the state of astroglia in the brains of transgenic gp120-tg mice treated with TDF, vibratome sections were immuonstained for GFAP with the addition of primary antibodies specific for GFAP and secondary antibodies conjugated to HRP that were reacted with NovaRed. In the hippocampus, as expected there was increased staining for GFAP in GP120 mouse brains compared to the wild type (Fig. 3a). The GFAP signal was also increased in wt treated with TDF compared to vehicle treated. However, there was no additive or synergistic increase in the gp120-tg mice (Fig. 3a). These observations are consistent with optical density measurements, which show a significant increase in GFAP density in GP120 mouse hippocampi compared to wild type (Fig. 3b). In the wt group, treatment with TDF led to a significant increase in GFAP optical density compared to the vehicle treated group, but this TDF-mediated increase was not seen in the GP120-tg group (Fig. 3b). To determine if TDF or gp120 affect proliferation of GFAP+ astroglia, GFAP+ cells were counted in the hippocampi using Image J. The number of GFAP+ cells in gp120-tg and gp120 + TDF mice was significantly increased compared to wt and to wt +TDF (Supplementary Fig. 3a). When analyzed by t test, the number of GFAP+ cells in wt + TDF was significnantly increased compared to wt mice.

Astroglia play a critical role in neuronal homeostasis and energy provision. To investigate if astroglial activation in GP120-tg mice is associated with alterations in transcription factor A, mitochondrial (TFAM), vibratome sections were double immunolabeled for TFAM (red) and GFAP (green). The signal for TFAM was noticeably decreased in hippocampi of gp120 tg, TDF-treated wt and TDF-treated gp120-tg mice compared to vehicle-treated wt mice (Fig. 3c). As expected, signal for GFAP was increased in gp120-tg mice whether treated with vehicle or TDF (Fig. 3c). GFAP signal was also more prominent in the TDF treated wt mice compared to vehicle treated wt mice (Fig. 3c). The merged images of GFAP and TFAM staining show an increase in yellow signal, indicating increased colocalization of TFAM with GFAP signal in all three groups compared to vehicle-treated wt mice (Fig. 3c). The pixel intensity for GFAP was significantly increased in gp120-tg mice and in TDF-treated mice compared to vehicle treated mice in both GP120 and wild type groups (Fig. 3d). Although TFAM signal decreased in tenofovir treated groups compared to vehicle treated groups, % colocalization of GFAP-TFAM increased significantly (Fig. 3e). An increase in colocalization of TFAM and GFAP concurrent with an increase in GFAP staining suggests a greater consumption of energy by astroglia concurrent with astrogliosis in the GP120 mice and mice treated with TDF.

### IBA1 is decreased in hippocampus of mice treated with TDF

**Figure 4:**
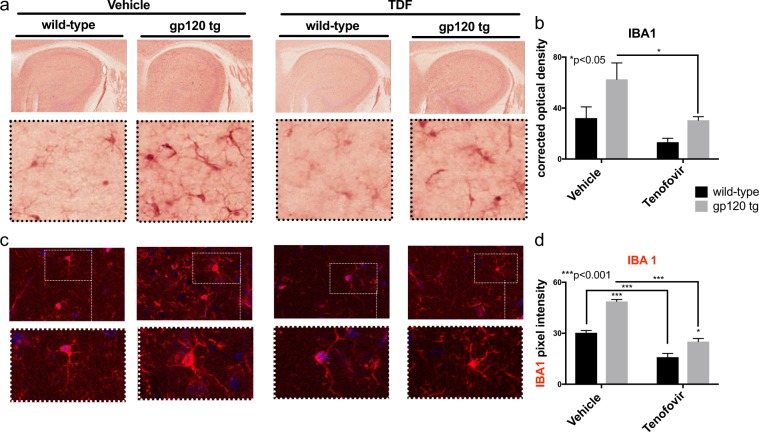
Tenofovir disproxil fumarate reduces IBA-1 signal in hippocampi of wt and gp120tg mice. Vibratome sections were immunolabelled for IBA1. (**a**) IBA1-DAB immunostaining in hippocampi. (**b**) Quantification of IBA1 corrected optical density. (**c**) IBA1+ glia (red) signal in hippocampi and quantification of IBA pixel intensity (**d**). Analyzed with two-way ANOVA test (wt, n = 3; wt + TDF, n = 4; gp120-tg, n = 4; gp120-tg, n = 4).

Ionized calcium binding adaptor molecule 1 (IBA1) is a calcium binding protein specific to microglia/macrophage that has actin-bundling activity and participates in phagocytosis and membrane ruffling in activated microglia (Ohasawa *et al*., 2004). Expression of IBA1 is upregulated in activated microglia in several brain diseases. To determine if microglia are activated in TDF-treated mice and in gp120-tg with and without treatment with TDF, we immunostained vibratome sections for IBA1 using NovaRed and preformed intensity/area analysis. IBA1 staining was increased in gp120-tg mouse brains compared to wild type brains in matched treatment samples (Fig. 4a). Treatment with TDF decreased the signal for IBA1 in both wt and gp120-tg mouse hippocampal regions (Fig. 4a). Quantification of IBA1 signal intensity/area revealed a significant increase in IBA1 in gp120-tg mouse brains compared to wt (Fig. 4b). IBA1 signal in hippocampi of TDF-treated wt mice was significantly reduced compared to vehicle treated wt mice (Fig. 4b). Similarly, IBA1 signal in the TDF-treated gp120-tg mice was significantly reduced compared to IBA1 in the gp120-tg group (Fig. 4b). To determine if TDF or gp120 affect proliferation of microglia, IBA1+ cells were counted in the hippocampi. The number of IBA1+ cells in gp120-tg mice was significantly increased compared to wt and to wt + TDF and gp120-tg TDF (Supplementary Fig. 3b).

To confirm the findings from chromagen immunostaining, vibratome sections of mouse brain were immunostained for IBA1, labeled with fluorescent-conjugated secondary antibodies and visualized using confocal microscopy in the hippocampi (Fig. 4c). The results mirrored the results from the NovaRed with a few exceptions. IBA1 signal was strongest in the vehicle-treated gp120-tg group, but TDF reduced this signal (Fig. 4d). Treatment with TDF also decreased the signal of IBA1 in both the wt mouse group, and, unlike the NovaRed staining, this decrease reached significance (Fig. 4d). These data suggest that IBA1 is upregulated in GP120-tg mouse brains and that this upregulation was blocked by treatment with TDF. The upregulation of IBA1 could suggest an increase in activated microglia in GP120-tg mice, which can be inhibited with TDF administration.

### TDF reduces levels of mRNA transcripts for inflammatory cytokines in gp120-tg mice

**Figure 5:**
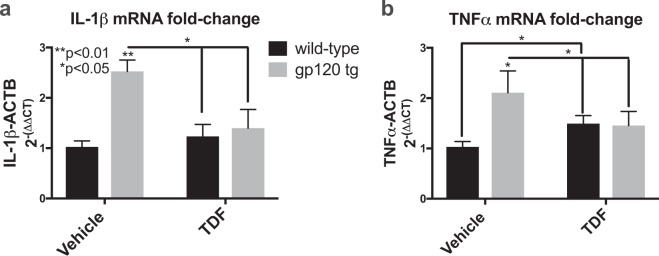
Tenofovir disoproxil fumarate reduces IL-1β and TNFα mRNA levels in gp120-tg brains. Total RNA was isolated from frontal cortex and analyzed by rt^2^PCR for IL-1β and TNFα mRNA levels. (**a,b**) Fold-change of IL-1β and TNFα mRNA compared to wt mice. Analyzed with two-way ANOVA test (wt, n = 3; wt + TDF, n = 3; gp120-tg, n = 3; gp120-tg, n = 3).

To determine relative levels of IL-1β and TNFα mRNA expression in the brains of wt and gp120-tg mice, total RNA was isolated from mouse brains from each group. IL-1β mRNA levels were increased 2.5-fold (**p < 0.01) in brains of gp120-tg mice compared to wt mice (A). However, in gp120-tg mice treated with TDF, levels of IL-1β mRNA were not significantly different than wt or wt treated with TDF (Fig. 5a). In wt mice, TDF increased TNF-α mRNA by 50% compared to wt mice treated with vehicle (**p < 0.05 by t-test) (Fig. 5b). TNFα mRNA levels were increased 2-fold (*p < 0.05) in brains of gp120-tg mice compared to wt mice (Fig. 5b). However, in gp120-tg mice treated with TDF, levels of TNFα mRNA were not significantly different than wt or wt treated with TDF (Fig. 5b). These data suggest a novel mechanism through which TDF simultaneously affects mitochondrial function and gliosis while reducing inflammatory signaling in the brain.

### TDF increases mitochondrial activity in human astroglial cultures

**Figure 6:**
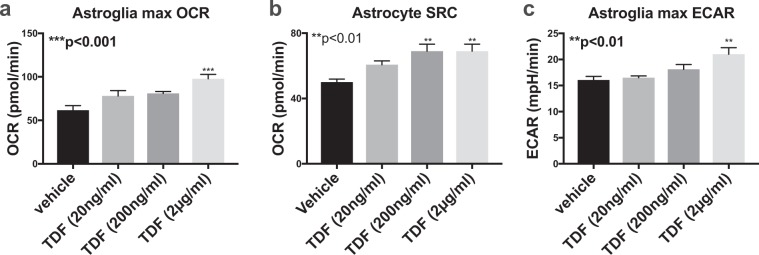
TDF increases mitochondrial activity in human astroglial cultures Human astroglia were treated with increasing doses of Tenofovir for 24 hours and then analyzed for OCR and ECAR using the Seahorse XF96 analyzer. (**a**) OCR in astroglia exposed to increasing doses of TDF. (**b**) Spare respiratory capacity was calculated by subtracting the basal OCR from the maximum OCR. (**c**) ECAR in astroglia exposed to increasing doses of TDF. Analyzed with one-way ANOVA test (n = 3/group).

The effects of ART drugs on astrglial mitochondrial function is an unexplored area of research. To determine how TDF affects mitochondrial activity in human astroglia, we exposed primary human astroglia to increasing doses of TDF (20 ng/ml, 200 ng/ml and 2 ug/ml) and measured oxygen consumption rate (OCR) and extracellular acidification rate (ECAR) using the Seahorse XF96 platform. Spare respiratory capacity (SRC) was calculated by subtracting the minimal OCR after oligomycin treatment from the maximum OCR after FCCP treatment. TDF caused a dose dependent increase in astroglial OCR with the highest dose increasing OCR by 50% compared to vehicle treatment (Fig. 6a). TDF also caused a dose-dependent increase in SRC with the two highest TDF doses being ~40% higher than vehicle treatment alone (Fig. 6b). The highest dose of TDF caused an increase in ECAR that was significantly higher than vehicle or the lower two doses (Fig. 6c). These data are consistent with the *in vivo* findings that show TDF increases TFAM expression in astroglia indicating increased mitochondrial activity. Data were analyzed using one-way ANOVA and Tukey’s multiple comparisons. Data are representative of at least three independent experiments in human primary astroglia from at least two independent genetic backgrounds.

### TDF blocks inflammatory gene expression in astroglia

**Figure 7:**
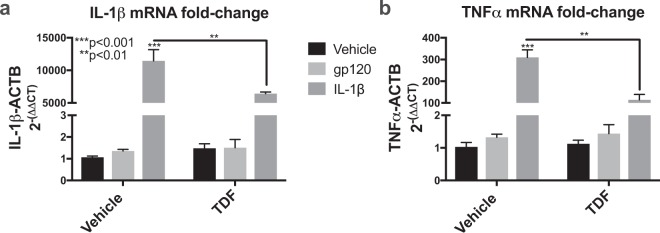
Tenofovir disoproxil fumarate reduces IL-1β -mediated increases in IL-1β and TNFα mRNA in human astroglia. Human astroglia were exposed to TDF (200 ng/ml), recombinant gp120 (100 ng/ml), or IL-1β (20 ng/ml) for 6 hours and then RNA was isolated. (**a**) Fold-change of TNFα mRNA normalized to ACTB mRNA. (**b**) Fold-change of IL-1β mRNA normalized to ACTB mRNA. Data were analyzed by two-way ANOVA (n = 3/group).

To understand how TDF affects inflammatory gene expression, astroglia were treated with gp120 (100 ng/ml) or IL-1β (20 ng/ml) with or without TDF (200 ng/ml). RNA was isolated and analyzed by real-time (rt^2^) pcr for fold change of IL-1β and TNFα mRNA transcripts. TDF and gp120 alone had little effect on levels of IL-1β or TNFα mRNA. However, IL-1β, as expected caused a robust increase in IL-1β mRNA levels, but co-treatment with TDF reduced this by 50% (***p < 0.001) (Fig. 7a). The HIV protein gp120 also caused an increase in astroglial IL-1b as expected (Fig. 7a). Similarly, IL-1β significantly increased TNFα mRNA levels, but co-treatment with TDF reduced levels by 60% (Fig. 7b). These data suggest that TDF is blocking inflammatory signaling in astroglia, consistent with what was observed in the mouse brains. Data were analyzed using one-way ANOVA and Tukey’s multiple comparisons. Data are representative of three independent experiments in one line of human primary astroglia. However, it is noteworthy that TDF did not reduce IL-1β or TNFα in a second culture of human primary astroglia of different genetic background, which responded similarly to IL-1β.

### Tenofovir disoproxil fumarate does not reduce IL-1β -mediated increases in IL-1β and TNFα mRNA in a human microglial cell line (HMC3)

**Figure 8:**
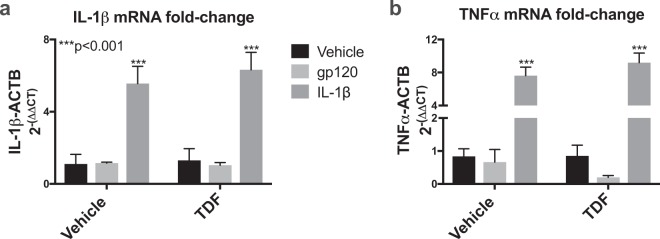
Tenofovir disoproxil fumarate does not reduce IL-1β -mediated increases in IL-1β and TNFα mRNA in a human microglial cell line (HMC3). HMC3 cells were exposed to TDF (200 ng/ml), recombinant gp120 (100 ng/ml), or IL-1β (20 ng/ml) for 6 hours and then RNA was isolated. (**a**) Fold-change of TNFα mRNA normalized to ACTB mRNA. (**b**) Fold-change of IL-1β mRNA normalized to ACTB mRNA. Data were analyzed by two-way ANOVA (n = 3/group).

To understand how TDF affects inflammatory gene expression in microglia, HMC3 cells were treated with gp120 (100 ng/ml) or IL-1β (20 ng/ml) with or without TDF (200 ng/ml). RNA was isolated and analyzed by rt^2^PCR for fold change of IL-1β and TNFα mRNA transcripts. TDF and gp120 alone had little effect on levels of IL-1β or TNFα mRNA. However, IL-1β as expected caused a robust increase in IL-1β and TNFα mRNA levels, but co-treatment with TDF had no effect on IL-1β levels (Fig. 8a) or TNFα levels (Fig. 8b). Data were analyzed using one-way ANOVA and Tukey’s multiple comparisons. Data are representative of at least three independent experiments.

## Discussion

This study provides the first evidence for the ART drug, TDF, affecting peripheral nerve function and CNS inflammation and mitochondrial biogenesis in an *in vivo* setting. TDF treatment produced large fiber MNCV slowing and small fiber-mediated paw thermal hyperalgesia in both wt and gp120-tg mice and in the CNS led to robust reductions in overall levels of a transcription factor that is crucial to maintaining mitochondrial networks, TFAM. Upon further investigation, the reductions in TFAM were localized to neurons, while TFAM was increased in astroglial cells in TDF-treated and gp120-tg mice. TDF induced astrogliosis^49^ in the mouse brains and in human astroglial cells in culture along with increased mitochondrial function. Interestingly, TDF blocked inflammatory cytokine transcription in the gp120-tg mouse brains and in the human astroglial cells in culture and reduced microgliosis in the gp120-tg mice. These potentially neuroprotective actions coincided with prevention of loss of hippocampal neurons in gp120-tg mice and protection of improved rotarod performance over time, an assay that can serve as an index of learning and memory^46,47^. This is consistent with clinical reports that initiating ART improved cognitive performance in cohort of individuals with HAND^50,51^. These findings suggest novel multiple complex activities of TDF in the CNS to alter mitochondrial biogenesis and inflammatory signaling and activation in astroglia and microglia.

Despite the persistently high prevalence of HIV-associated neurological disorders in PWH on suppressive ART, relatively few studies have investigated how modern ART drugs affect the peripheral and central nervous systems. A prior study reported that the dideoxynucleoside antiretroviral drug didanosine caused loss of small sensory intra-epidermal nerve fibers (IENF) when given to young gp120-tg mice that appeared to represent acceleration of a slowly developing distal degenerative sensory neuropathy^45^. However, while didanosine did not impact nerve structure, electrophysiology or sensory function in wt mice, we found TDF produced both marked large motor fiber NCV slowing and paw thermal hyperalgesia in both gp120-tg and wt mice. The 50 mg/kg/day dose of TDF we used was selected from prior pharmacokinetic and toxicity studies in mice^16,52,53^ that by allometric scaling^54,55^ equates to a human dose of 0.42 mg/kg/day or 29 mg/70 kg person, well within the clinical dose of 300 mg/day. There are reports of pain and other manifestations of peripheral neuropathy in some patients treated with TDF^56–58^ that have been linked to mitochondrial toxicity^20,59^. Mitochondrial dysfunction is increasingly implicated in the pathogenesis of a variety of peripheral neuropathies^60,61^. However, separating HIV vs ART drug induced peripheral neuropathy has been a controversial undertaking in clinical research. Our finding that unlike didanosine^45^, TDF also produced peripheral neuropathy in wt mice may represent a model system for evaluating mechanisms of pure ART neurotoxicity.

A study of 15 ART drugs found variable levels of neurotoxicity, with TDF reducing both MAP2 immunostaining in neuronal cultures and also the signal emanating from neurons after exposure to a mitochondrial potential sensitive dye^20^. Our data corroborate these findings in an *in vivo* setting by showing alterations in MAP2 signal and transcription factors that regulate mitochondrial biogenesis. In a study that utilized the SIV-infected pig tailed macaque, an ART cocktail including TDF caused accumulation of reactive oxygen species and neurotoxicity, though these events were not linked directly to mitochondrial dysfunction^62^. The findings in this report are also consistent with a recent study that showed TDF reduces neurogenesis in the CNS of mice^16^. Xu *et al*. used a combination of ART drugs to determine the effects on neural precursor cells using *in vivo* and *in vitro* models. The authors concluded that ART, mainly driven by TDF, may exacerbate brain injuries and neurocognitive impairment associated with HAND by affecting neurogenesis. The study by Xu *et al*. did not investigate mitochondrial activity or inflammatory signaling but does offer support for the premise that ART can affect CNS function^16^. The findings presented here do not explain how TDF protected performance on a rotarod over time as reported in these studies. TDF effects on neurons may be context dependent, affecting different pathways in a healthy brain versus HIV+ brain. As discussed below, these differential effects of TDF may also be related to the observations in this study showing effects that TDF has on astroglia, microglia, and inflammatory gene expression in the brain. Future studies are needed to better understand the impact of TDF on neuronal survival in hippocampi.

Several pharmacogenetic studies have provided indirect evidence for ART-associated mitochondrial dysfunction leading to neurotoxicity. A study of the Adult AIDS Clinical Trials Group (ACTG) uncovered an association between mitochondrial haplogroup T and NRTI-associated peripheral neuropathy^5^. A separate study reported that mitochondrial polymorphism 4918 G may confer increased risk for peripheral neuropathy in people on ART^63^. Polymorphisms in the P2X4R and CAMKK2 genes may be associated with HIV-SN, and increased TNFα expression^64^. These findings may provide further evidence that inflammatory processes can compromise mitochondrial function in neurons^65^. Moreover, chemokine receptors on cells enveloping cutaneous nerves may facilitate infiltration of immune cells in patients with HIV-SN^66^. Recent studies suggest that immunometabolic mechanisms in microglia and astroglia may play a similar role in HAND^40,67,68^. While TDF reduced inflammatory gene transcription in the mouse brain and in human astroglia, increased TFAM in astroglia may suggest similar increased metabolic function in this cell type. Hence, these studies show that mitochondrial biogenesis, or more specifically TFAM, may be a therapeutic target for ART and HIV-induced neurological disorders. The reductions in TFAM in neurons in TDF-treated and gp120-tg mice coincide with reduced MAP2 signal and increased TFAM in astroglia. However, data show that TDF and gp120 do not act on TFAM in a synergistic or additive manner, suggesting that a mechanism may be in place to prevent further down regulation of TFAM. The fact that PGC-1α was increased in the brain may indicate that TDF and gp120 interfere with TFAM pathway downstream of PGC-1α. The HIV protein gp120 has been shown to activate p38 kinase^69^, which can activate mitochondrial biogenesis^70,71^, possibly explaining the high levels PGC-1α in the gp120-tg mice. These studies also confirmed previous findings of reduced DNM1L^41^, which was partially reversed by TDF. While gp120-tg mice showed no indices of peripheral neuropathy, including first test rodarod performance, they did show a failure to improve rotarod performance upon repeated testing, suggestive of cognitive impairment in these mice that may be caused by mitochondrial alterations and inflammatory gene expression.

The question remains as to whether the effect of TDF on neuronal mitochondrial function is direct or mediated through activation of microglia and astroglia. Previous studies suggest that TDF can affect neurons directly^16,72^. Our data clearly show that TDF has robust effects on glial activation both *in vivo* and *in vitro*. These data are consistent with studies by Cohen *et al*. that showed ART can induce inflammatory gene expression and cellular senescence in astroglia and endothelial cells^72,73^. Therefore, we hypothesize that TDF affects neuronal mitochondrial function directly and also through activation of glia. Indirect effects on neuronal mitochondrial function through activation of astroglia are consistent with our recent finding in HAND brains that astroglial TFAM is increased and neuronal TFAM is decreased^37^. This may be explained by a mechanism in which activated astroglia sequester energy substrate that would otherwise be utilized by neurons^74^. Another possibility is that astroglia secrete neurotoxic molecules as has been found using *in vitro* models^67,75^. Understanding the signaling between neurons and astroglia in states of chronic inflammation will be important to determine the role of TDF on neuronal mitochondrial function. The effects of TDF on TFAM and DNM1L deserve further investigation in human samples and *in vitro* and *in vivo* models.

HIV and ART may affect the phenotype of astroglia and microglia. The current model of HIV-induced CNS injury involves infiltration of infected monocytes into the brain where they secrete virus, viral proteins and inflammatory cytokines^76,77^. The virus can infect perivascular macrophages which secrete more toxic molecules initiating a neurotoxic cascade involving activation of uninfected bystander microglia and astroglia^78,79^. Although controversial, some studies suggest that astroglia are infected by HIV and express viral proteins at a low level^80–82^. With the advent of ART, it is believed that the viral component of the neurotoxicity is reduced, but how ART affects microglia and astroglia is not well understood. Moreover, recent findings suggest that activity of microglia and astroglia place them within a spectrum of phenotypes ranging from pro-inflammatory to anti-inflammatory^75,83,84^. Microglia and astroglia assume varying phenotypes in response to specific types of brain injury^85–87^. Modulating these phenotypes is a potential therapeutic strategy for neurodegenerative diseases. This study suggests that TDF, and possibly other reverse transcriptase inhibitors, may modulate gene expression networks and activation states of microglia and astroglia. Consistent with our findings, Melchjorsen *et al*. found that tenofovir and zidovudine each affect inflammatory cytokine production in human monocytes, though in different manners with tenofovir altering inflammatory gene expression after stimulation with ligands for toll-like receptor^88^. The data presented here suggest a dynamic relationship between TDF and gliosis. In wt mice, TDF clearly activates astroglia and induces a significant increase in TNFα mRNA, both of which have been associated with HIV-infected brains that were exposed to ART^40^. The more unexpected finding was that TDF reduced the signal for IBA1 protein, numbers of IBA1+ cells and mRNA levels for TNFα and IL-1β in gp120-tg mice. These findings suggest that TDF effects on inflammatory signaling in the brain may be different in an inflamed environment compared to steady state. However, these data may be consistent with a finding in postmortem brain specimens from HIV+ people on ART that showed regimens including TDF were associated with reduced Aβ plaque deposition in the brain^17^. The robust reduction of microglial activation by TDF in the gp120-tg mice may indeed indicate that in the context of HIV protein expression, TDF may steer astroglia and microglia toward a more anti-inflammatory phenotype, which could have implications for therapeutic strategies. This reduction in microglial activation and expression of inflammatory cytokines could partially explain the results presented here showing that TDF administration improves rotarod performance in the gp120-tg mice. Further studies are needed to determine the effect of TDF on microglial and astroglial function and phenotypic markers and the downstream effects on cognitive function in the context of neuroinflammation.

Since the gp120-tg model expresses gp120 from the GFAP promoter, the initial inflammatory signal in this model may be from a combination of inflammatory gene expression from astroglia stimulated by endogenous gp120 expression and in microglia stimulated by exogenous gp120 and cytokines from astroglia. Therefore, it is important to understand if TDF is blocking IL-1β and TNFα mRNA transcription in the astroglia or microglia to understand the observed *in vivo* inhibition. Further, it is important to understand if this finding may be translatable to human brain cells. While the human astroglial and microglial models are not without limitations, the findings that TDF reduced IL-1β-induced IL-1β and TNFα transcription in human astroglia are consistent with the findings in the mouse brains. However, TDF did not have this anti-inflammatory effect in another line of astroglia with different genetic background suggesting that TDF effects on inflammation may vary by individual or population due to genetic interactions. The fact that TDF did not have the same effect on the HMC3 cells suggests that in the mice, TDF is acting on the astroglial inflammatory gene expression and that is preventing activation of microglia. Differences in the experimental design between the *in vivo* and *in vitro* studies could account for the different effects of TDF on microglial cells. The mice were exposed to TDF for four weeks, compared to 24 hours in the *in vitro* experiments using human cells. Increasing doses of TDF were used to determine responses by glial cells. However, the highest dose used (2ug/ml) is likely not relevant to the human condition and the results should be considered in that context. This novel mechanism of TDF interactions with astroglial and microglial activation and gene expression may have implications for ART regimens affecting glial phenotype in people with neurological disorders.

The major limitations of this study include a relatively small sample size, use of only one ART drug, the use of *in vitro* models that are known to not exactly replicate the biology of brain cells *in vivo* and the use of the a mouse model that expresses one HIV protein from a promoter active only in astroglia. The issue of small sample size is somewhat offset by the robust differences in readouts from different levels of analysis, across different model systems and species. These findings should be further investigated to better understand how TDF affects astroglia and the downstream effects on neuronal function. The gp120-tg mouse model for HIV-induced neurotoxicity is not ideal for modeling HIV infection of the brain, but it does present many of the neuropathological features found in postmortem HIV+ brain tissues^41,42,44,89^, along with some aspects of cognitive impairment (d’Hooge *et al*. 1999; Maung *et al*. 2014) that were reflected in the failure to improve rotarod performance by these mice in the present study. This finding suggests that cognitive impairment manifests in this model by 9 months of age, coinciding with loss of hippocampal neurons. In future studies, it will be important to determine the levels of PGC1α in neuron and glial cells. It will also be important in a future study to investigate TFAM alterations in microglial cells, as metabolic alterations occur in myeloid lineage cells during inflammatory processes. Moreover, more investigation of the effect of TDF on neuronal cells is needed to fully understand the implications of the findings presented here.

These findings identify peripheral and central neurotoxicity of TDF in wt and gp120-tg mice in conjunction with protection against loss of hippocampal neurons and impaired learning/memory. Novel functions of TDF in modulating inflammatory signaling and mitochondrial homeostasis in the brain were also identified. Also clear from this report is the role of astroglia in responding to ART drugs and the downstream effects on inflammatory signaling. Monitoring ART drugs effects on brain cells may lead to optimization of ART regimens and reduction of neuropsychiatric adverse events in PWH. Furthermore, astroglia may represent a promising target for reducing microgliosis and inflammatory gene expression.

## Supplementary information

Supplementary Dataset 1

## Data Availability

All reasonable requests for data or materials will be provided in a timely manner.
