# Supplementary material for: Tenofovir disoproxil fumarate induces peripheral neuropathy and alters inflammation and mitochondrial biogenesis in the brains of mice
Source: Sci Rep. 2019 Nov 20;9:17158. doi: 10.1038/s41598-019-53466-x (PMC6868155; doi:10.1038/s41598-019-53466-x)
Supplement: Supplementary file 1 — Supplementary Dataset 1 [file 41598_2019_53466_MOESM1_ESM.pdf]

Tenofovir disoproxil fumarate induces peripheral neuropathy and alters inflammation and mitochondrial biogenesis in the brains of mice

Jerel Adam Fields[a*#], Mary K. Swinton[a], Aliyah Carson[a], Benchawanna Soontornniyomkij[c], Charmaine Lindsay[c], May Madi Han[c], Katie Frizzi[c], Shrey Sambhwani[a], Anne Murphy[d], Cristian L. Achim[c], Ronald J. Ellis[b] and Nigel A. Calcutt[c#]

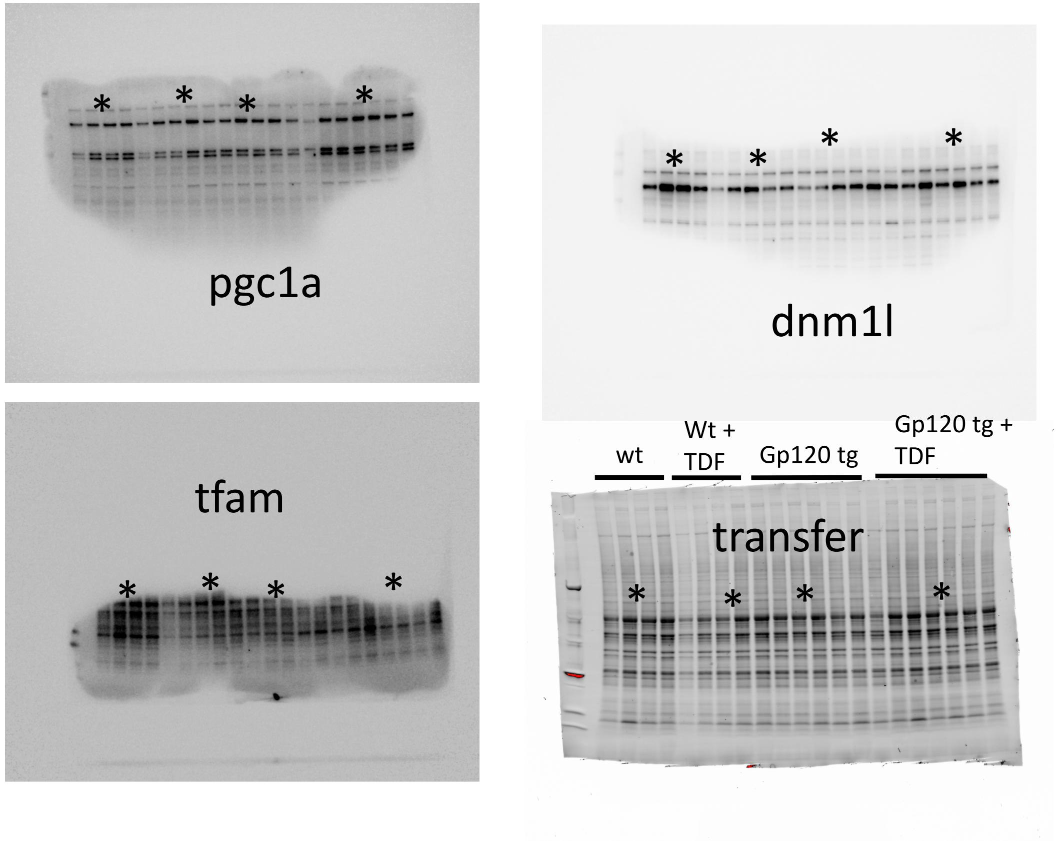
pgc1a
dnm1l
tfam
wt
Wt +
TDF
Gp120 tg
Gp120 tg +
TDF
transfer

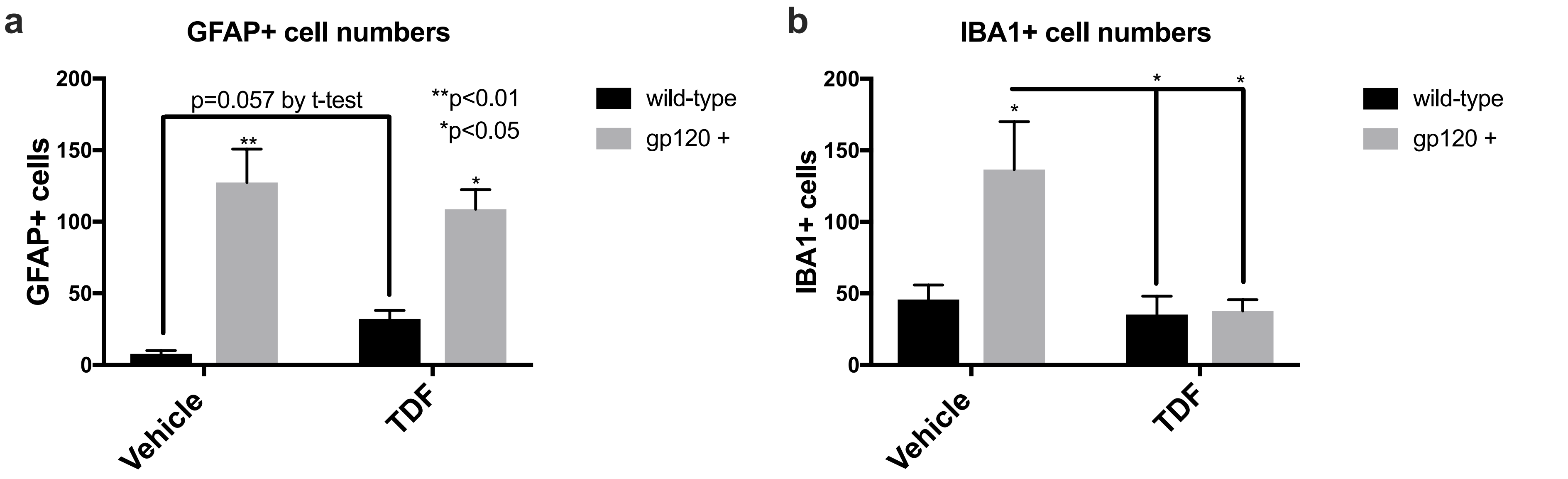

Supplementary Figure 2: The number of GFAP+ and IBA1+ cells in hippocampi were counted in the images from Figure 4, using Image J analysis software. GFAP+ cells were increased in gp120-tg mice compared to wt (**a**). However, the number of GFAP+ cells in wt mice that were treated with TDF were increased, though not significantly, compared to wt (**a**). IBA1+ cells were increased compared to wt (**b**). However, IBA1+ cells were reduced in wt and in gp120-tg mice that were treated with TDF (**b**). Data were analyzed by two-way ANOVA followed by Tukey’s multiple comparisons.
